# Diet high in fat and sucrose induces rapid onset of obesity-related metabolic syndrome partly through rapid response of genes involved in lipogenesis, insulin signalling and inflammation in mice

**DOI:** 10.1186/1758-5996-4-32

**Published:** 2012-07-04

**Authors:** Zhi-Hong Yang, Hiroko Miyahara, Jiro Takeo, Masashi Katayama

**Affiliations:** 1 Central Research Laboratory, Tokyo Innovation Center, Nippon Suisan Kaisha, Ltd., 32–3 Nanakuni 1 Chome Hachioji, Tokyo, 192-0991, Japan

**Keywords:** High fat–high sucrose diet, Hepatosteatosis, Insulin signalling, Inflammation

## Abstract

**Background:**

Frequent consumption of a diet high in fat and sucrose contributes to lifestyle-related diseases. However, limited information is available regarding the short-term effects of such a diet on the onset of obesity-associated metabolic abnormalities.

**Methods:**

Male C57BL/6 J mice were divided into two groups and fed a standard chow diet (control group) or a high fat–high sucrose diet containing 21% fat and 34% sucrose (HF–HS diet group) for 2 or 4 weeks.

**Results:**

The HF–HS diet significantly induced body weight gain beginning at week 1 and similarly increased mesenteric white adipose tissue weight and plasma insulin levels at weeks 2 and 4. Plasma resistin levels were notably elevated after feeding with the HF–HS diet for 4 weeks. Measurement of hepatic triglycerides and Oil Red O staining clearly indicated increased hepatic lipid accumulation in response to the HF–HS diet as early as 2 weeks. Quantitative PCR analysis of liver and white adipose tissue indicated that, starting at week 2, the HF–HS diet upregulated mRNA expression from genes involved in lipid metabolism and inflammation and downregulated genes involved in insulin signalling. Although plasma cholesterol levels were also rapidly increased by the HF–HS diet, no differences were found between the control and HF–HS diet–fed animals in the expression of key genes involved in cholesterol biosynthesis.

**Conclusions:**

Our study demonstrates that the rapid onset of hepatosteatosis, adipose tissue hypertrophy and hyperinsulinemia by ingestion of a diet high in fat and sucrose may possibly be due to the rapid response of lipogenic, insulin signalling and inflammatory genes.

## Background

Being overweight or obese is one of the leading risks for death worldwide. Increasing evidence has indicated that obesity is linked to numerous comorbidity diseases such as type 2 diabetes, hypertension, hypercholesterolaemia, hypertriglyceridaemia, and non-alcoholic fatty liver disease [1-3]. It is estimated that 20–25% of the world’s adult population has obesity-related metabolic syndrome, and they are twice as likely to die and three times as likely to have a heart attack or stroke compared with people without the syndrome [4,5]. Besides an obesogenic environment and reduced energy expenditure during work and leisure activities, one of the primary causes of the current epidemic of obesity and related metabolic disorders is related to the western-style diet, which includes excessive intake of high-fat and high-sucrose foods. Several studies have assessed the long-term (over 10 weeks ~ 2 years) effects of high-fat and/or high-sucrose diets on metabolic risk factors [6-8]. On the other hand, limited information is available about the short-term effects of a high fat–high sucrose (HF–HS) diet on the onset of hepatosteatosis and changes in the expression of genes involved in lipid and cholesterol metabolism, insulin signalling, and inflammation in liver and white adipose tissue (WAT).

Liver and WAT are important for metabolic regulation. As consequences of obesity, a fatty liver and adipocyte hypertrophy play crucial roles in the development of metabolic syndrome via multiple mechanisms including impaired insulin signalling and inflammation [9,10]. To better understand the influences of a western-style diet that is high in fat and sucrose on the onset of obesity-associated metabolic abnormalities, the present study was designed to assess the short-term (2 and 4 weeks) effects of a HF–HS diet on the risk factors for metabolic disruptions. Therefore, we investigated the underlying mechanism in C57BL/6 J mice, an inbred mouse strain that has been used for studies of obesity and diabetes owing to its susceptibility to these diseases in response to a high-fat diet [11,12].

## Methods

### Animals

This study was approved by the Institutional Animal Care and Use Committee at Kitayama Rabesu Inc. (Nagano, Japan), where the animals were housed for the entire experimental period. Eight-week-old male C57BL/6 J mice (Charles River Laboratories, Inc., Kanagawa, Japan) were housed in a room at 23 ± 1 °C with a 12/12-h light–dark cycle.

### Diets

The mice were fed two different diets. The low fat–low sucrose diet was standard mouse chow CRF–1 (Oriental Yeast Co. Ltd., Tokyo, Japan). Its composition was (wt/wt): 5.4% fat, 53.8% carbohydrate, 21.9% protein, 2.9% fibre, 6.6% minerals, added vitamins A, D, and E, and 0.02% cholesterol (357 kcal per 100 g). The HF–HS diet was a milk fat–based diet (TD.88137; Harlan Laboratories Inc., Indianapolis, IN, USA). Its composition was (wt/wt): 21.2% fat, 49.1% carbohydrate (34.1% sucrose plus 15% corn starch), 17.3% protein, 5.0% fibre, 3.5% minerals, 0.4% CaCO_3_, 1% vitamin mix, 0.004% antioxidants, and 0.2% cholesterol. Fats provided 42% of the calories, and the diet yielded 450 kcal per 100 g.

### Experimental protocol

The animals had free access to water and standard mouse chow CRF–1 for an acclimatization period of 1 week. Thereafter, animals weighing 23–24 g were randomly assigned to two groups for the feeding experiment. The control group (n = 12) was fed standard mouse chow CRF–1, and the HF–HS group (n = 12) was fed the diet TD.88137, which was high in fat and sucrose. Body weight and food intake were monitored throughout the study. At the end of 2 weeks and 4 weeks, the mice (the control group, n = 6; HF–HS diet group, n = 6) were anesthetized with 4% sodium pentobarbital (Dainippon Sumitomo Pharma, Osaka, Japan). Whole blood was collected from the abdominal aorta into EDTA-coated tubes, and plasma was obtained by centrifugation at 3000 rpm for 15 min at 4 °C and stored at −80 °C until biochemical analysis. Liver and mesenteric WAT were dissected and weighed after a short wash in cold phosphate-buffered saline (pH 7.4). Organs were immediately flash-frozen in liquid nitrogen and stored at −80 °C until further lipid extraction and quantitative polymerase chain reaction (QPCR) analysis. Liver portions were fixed in 10% formalin for histological examination.

### Plasma biochemical analysis

Plasma triglycerides, free fatty acids, total cholesterol, and high-density lipoprotein cholesterol (HDL–C) were determined enzymatically using commercially available reagent kits (Wako Pure Chemical Industries, Ltd., Osaka, Japan). Low-density lipoprotein cholesterol (LDL–C) was calculated as total cholesterol − HDL-triglyceride × 0.2.

Plasma concentrations of insulin and adipokines, which have wide-ranging effects on energy intake/expenditure as well as on carbohydrate and lipid metabolism, were measured by enzyme-linked immunosorbent assays (ELISA). Mouse Insulin ELISA kit (Morinaga Institute of Biological Science, Inc., Yokohama, Japan), Mouse Adiponectin ELISA kit (Otsuka Pharmaceutical Co., Ltd., Tokyo, Japan), and Mouse Resistin ELISA kit (Shibayagi Co. Ltd., Gunma, Japan) were used to determine plasma levels of these proteins.

### Extraction and analysis of hepatic lipid content

Extraction of total lipids from a portion of liver was performed as described by Folch *et al.*[13] in the presence of butylated hydroxytoluene as an antioxidant. Extracted lipids were dried using a vacuum concentrator (Concentrator Plus 5305, Eppendorf) and dissolved in 2-propanol. Triglyceride and total cholesterol concentrations were determined using commercial enzyme kits (Wako). For hepatic histology, formalin-fixed liver tissue was embedded in paraffin, cut into 10-μm sections, and stained with hematoxylin and eosin. Oil Red O (Sigma Aldrich, St. Louis, MO, USA) staining of neutral lipids was done on frozen sections.

### QPCR

Total RNA was extracted from frozen liver and WAT using the SV Total RNA Isolation System (Promega Corp., Madison, WI, USA) according to the manufacturer’s instructions. RNA concentration and purity were assessed based on absorbance at 260 nm and 280 nm. First-strand cDNA was synthesized from total RNA using a PrimeScript II 1st strand cDNA Synthesis kit (TaKaRa Bio, Otsu, Japan) using oligo dT-adaptor primers and 1 μg total RNA as the template. The resulting cDNA was used for QPCR amplification in a 96-well format with SYBR Premix Ex Taq (TaKaRa Bio) and a 7500 Real-Time PCR System (Life Technologies Co., Japan). Expression levels of test genes were normalized to the expression of the housekeeping gene encoding 18 S ribosomal RNA. The primers used for QPCR are listed in Table 1.

**Table 1:** Primers used for QPCR

**Gene**	**Forward primer (5′ to 3′)**	**Reverse primer (5′ to 3′)**
*AMPK*	AAGATCGGACACTACGTCCTG	TGCCACTTTATGGCCTGTCAA
*Akt2*	ACGTGGTGAATACATCAAGACC	ACCCAATGAAAGATCCATCACTC
*CD36*	ATGGGCTGTGATCGGAACTG	AGCCAGGACTGCACCAATAAC
*CD68*	GGACCCACAACTGTCACTCAT	AAGCCCCACTTTAGCTTTACC
*FAS*	GGAGGTGGTGATAGCCGGTAT	TGGGTAATCCATAGAGCCCAG
*HMGCR*	TGTTCACCGGCAACAACAAGA	CCGCGTTATCGTCAGGATGA
*IRS2*	TCTACACCCGAGACGAACACT	TGGGCCTTTGCCCGATTATG
*LDLR*	TGACTCAGACGAACAAGGCTG	ATCTAGGCAATCTCGGTCTCC
*LPL*	TTGCCCTAAGGACCCCTGAA	ACAGAGTCTGCTAATCCAGGAAT
*LXRα*	CTCAATGCCTGATGTTTCTCCT	TCCAACCCTATCCCTAAAGCAA
*MAC1*	ATGGACGCTGATGGCAATACC	TCCCCATTCACGTCTCCCA
*MMP3*	ACATGGAGACTTTGTCCCTTTTG	TTGGCTGAGTGGTAGAGTCCC
*PPARγ*	GGAAGACCACTCGCATTCCTT	GTAATCAGCAACCATTGGGTCA
*SCD-1*	TTCTTGCGATACACTCTGGTGC	CGGGATTGAATGTTCTTGTCGT
*SREBP1c*	GATGTGCGAACTGGACA	CATAGGGGGCGTCAAACAG
*SREBP2*	TGGGCGATGAGCTGACTCT	ACTGTAGCATCTCGTCGATGT

*AMPK*: 5' adenosine monophosphate-activated protein kinase, *Akt2*: protein kinase B beta, *CD36*: CD36 antigen, *CD68*: CD68 antigen, *FAS*: fatty acid synthase, *HMGCR*: 3-hydroxy-3-methylglutaryl-coenzyme A reductase, IRS2: insulin receptor substrate 2, *LDLR*: low-density lipoprotein receptor, *LPL*: lipoprotein lipase, *LXRα*: liver X receptor alpha, *MAC1*: macrophage-1 antigen, *MMP3*: matrix metalloprotease 3, *PPARγ*: peroxisome proliferator activated receptor gamma, *SCD-1*: stearoyl CoA desaturase-1, *SREBP*: sterol regulatory element binding protein.

### Statistical analysis

The data are presented as the mean ± SE. All statistical tests were performed using the Student's *t*-test, and statistical significance was considered as *P* < 0.05.

## Results

### Body, WAT, and liver weights

**Figure 1:**
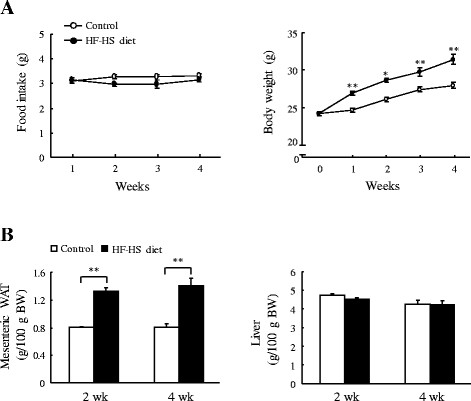
**Changes in body, white adipose tissue (WAT), and liver weights.** Food intake and body weight (**A**) and mesenteric WAT and liver weights (**B**) are shown. HF–HS diet: high fat–high sucrose diet. C57BL/6 J mice were fed standard chow diet or the HF–HS diet (milk fat 21% and sucrose 34%) for 2 or 4 weeks. Values are the mean ± SE, n = 6. *P < 0.05; **P < 0.01.

Mice in the HF–HS diet group gained weight rapidly. As shown in Figure 1, the HF–HS diet increased the body weight by 8.3% (*P* < 0.01), 9.1% (*P* < 0.05), 7.8% (*P* < 0.01), and 11.4% (*P* < 0.01) at the end of weeks 1, 2, 3, and 4, respectively, although food intake remained unchanged in the two groups over the 4 weeks. Compared with the control group fed standard chow diet, mesenteric WAT weight in the HF–HS diet group increased by 64.9% and 74.2% (*P* < 0.01 for both) at the end of weeks 2 and 4, respectively. There were no differences in liver weight.

### Plasma lipids profile

**Figure 2:**
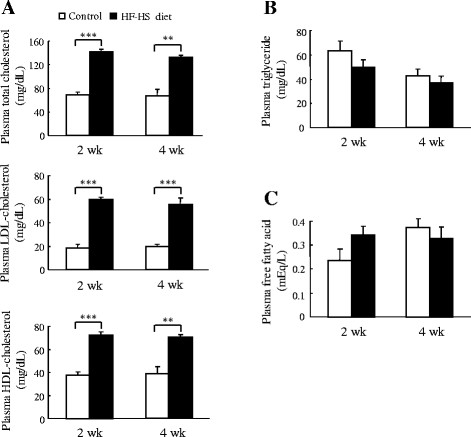
**Effect of the HF–HS diet on the plasma lipid profile.** Plasma levels of cholesterol (total cholesterol, LDL–cholesterol, and HDL–cholesterol) (**A**), triglycerides (**B**), and free fatty acids (**C**) are shown. HF–HS diet: high fat–high sucrose diet. C57BL/6 J mice were fed standard chow diet or the HF–HS diet (milk fat 21% and sucrose 34%) for 2 or 4 weeks. Values are the mean ± SE, n = 6. ** *P* < 0.01; *** *P* < 0.001.

As shown in Figure 2, the HF–HS diet increased total cholesterol by 103.8% (*P* < 0.001) and 97.1% (*P* < 0.01) at the end of weeks 2 and 4, respectively. LDL–C was increased by 218.6% and 175.5% (*P* < 0.001 for both) at the end of weeks 2 and 4, respectively. HDL–C was increased by 80.8% and 89.5% (*P* < 0.01 for both) at the end of weeks 2 and 4, respectively. However, there were no differences in the plasma triglyceride or free fatty acid levels between the control and HF–HS groups.

### Plasma levels of insulin and adipokines

**Figure 3:**
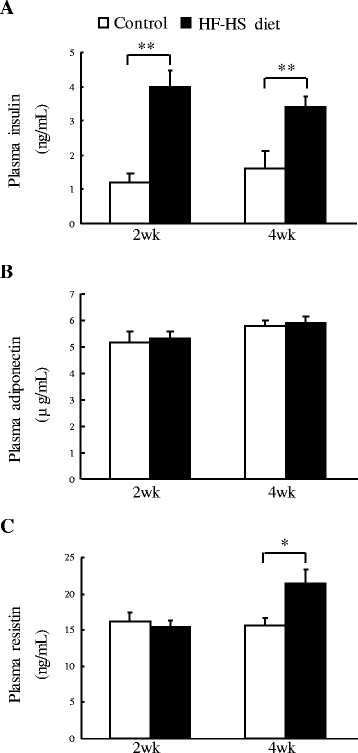
**Effect of the HF–HS diet on plasma levels of insulin and adipokines**. Plasma levels of insulin (**A**), adiponectin (**B**), and resistin (**C**) are shown. HF–HS diet: high fat–high sucrose diet. C57BL/6 J mice were fed standard chow diet or the HF–HS diet (milk fat 21% and sucrose 34%) for 2 or 4 weeks. * *P* < 0.05. ** *P* < 0.01.

As shown in Figure 3, the HF–HS diet caused 2.2-fold and 1.1-fold increases (*P* < 0.01 for both) in plasma insulin levels at the end of weeks 2 and 4, respectively, compared to the control group. Concerning plasma adipokine levels, there was an increase in plasma resistin of 38% (*P* < 0.05) at the end of week 4 but not at week 2 in the HF–HS diet group compared to the control group, whereas no difference in plasma adiponectin level was found between the control and the HF–HS groups at either time point.

### Lipid accumulation in liver

**Figure 4:**
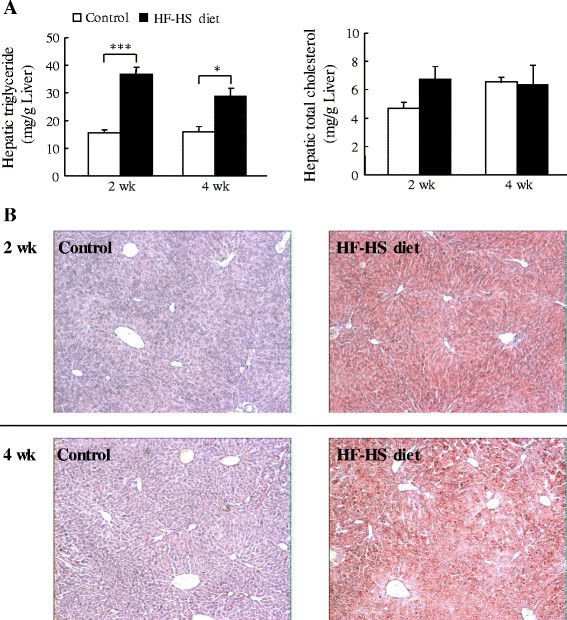
**Effect of the HF–HS diet on hepatic lipid accumulation.** (**A**) Hepatic triglyceride and total cholesterol levels. (**B**) Photomicrographs of liver sections stained with hematoxylin-eosin and Oil Red O at week 2 (upper panel) and week 4 (lower panel). HF–HS diet: high fat–high sucrose diet. C57BL/6 J mice were fed standard chow diet or the HF–HS diet (milk fat 21% and sucrose 34%) for 2 or 4 weeks. Values are the mean ± SE, n = 6. ** *P* < 0.01, *** *P* < 0.001.

Although the difference in hepatic cholesterol between animals fed the control and HF–HS diets did not reach statistical significance, the HF–HS diet elevated hepatic triglyceride levels by 135% (*P* < 0.001) at week 2 and 42% (*P* < 0.05) at week 4 (Figure 4A). Oil Red O staining of liver tissue also showed that the HF–HS diet caused notable lipid accumulation as early as week 2 (Figure 4B).

### Expression of key genes involved in lipid and cholesterol metabolism and insulin signalling in liver

**Figure 5:**
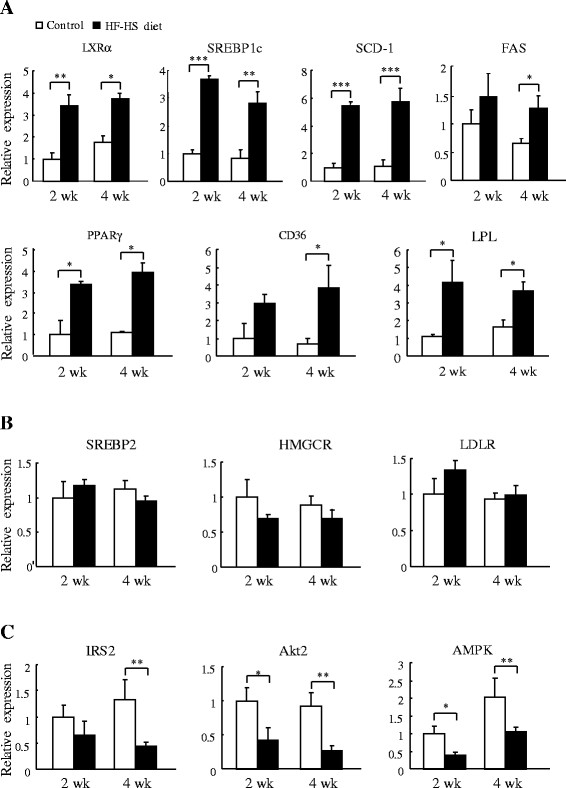
**Effect of the HF–HS diet on hepatic genes for lipid/cholesterol metabolism and insulin signalling.** (**A**) mRNA expression of genes related to lipid metabolism: *LXRa*, *SREBP1c*, *SCD-1*, *FAS*, *PPARγ*, *CD36*, and *LPL*. (**B**) mRNA expression of genes related to cholesterol metabolism: *SREBP2*, *HMGCR*, and *LDLR*. (**C**) mRNA expression of genes related to insulin signalling: *IRS2*, *Akt2*, and *AMPK*. HF–HS diet: high fat–high sucrose diet. C57BL/6 J mice were fed standard chow diet or the HF–HS diet (milk fat 21% and sucrose 34%) for 2 or 4 weeks. Values are the mean ± SE and are relative to control values, which were set at 1.0. n = 6. * *P* < 0.05, ** *P* < 0.01, *** *P* < 0.001.

The expression of genes closely involved in lipogenesis and lipid transport was rapidly upregulated in animals given the HF–HS diet (Figure 5A). At week 2 and week 4, respectively, the HF–HS diet increased the mRNA expression of liver X receptor alpha (*LXRα*) by 2.4 fold (*P* < 0.01) and 1.1 fold (*P* < 0.05), that of sterol regulatory element binding protein 1c (*SREBP-1c*) by 2.7 fold (*P* < 0.001) and 2.3 fold (*P* < 0.01), that of stearoyl CoA desaturase-1 (*SCD-1*) by 4.4 and 4.6 fold (*P* < 0.001 for both), that of fatty acid synthase (*FAS*) by 45% not significant and 96% (*P* < 0.05), that of peroxisome proliferator activated receptor gamma (*PPARγ*) by 2.4 fold and 2.6 fold (*P* < 0.05 for both), that of CD36 by 2.0 (not significant) and 4.5 fold (*P* < 0.05), and that of lipoprotein lipase (*LPL*) by 2.7 fold and 1.2 fold (*P* < 0.05 for both). In contrast, as shown in Figure 5B, the HF–HS diet did not alter the expression of several key genes involved in cholesterol biosynthesis, such as sterol regulatory element binding protein 2 (*SREBP-2*), 3-hydroxy-3-methyl-glutaryl-CoA reductase (*HMGCR*), and low-density lipoprotein receptor (*LDLR*). Furthermore, mRNA expression from genes involved in insulin signalling was downregulated by the HF–HS diet (Figure 5C). At week 2 and week 4, respectively, the HF–HS diet decreased the mRNA expression of insulin receptor substrate 2 (*IRS2*) by 35% (not significant) and 68% (*P* < 0.01), that of protein kinase B beta (*Akt2*) by 58% (*P* < 0.05) and 71% (*P* < 0.01), and that of 5' adenosine monophosphate-activated protein kinase (*AMPK*) by 60% (*P* < 0.05) and 42% (*P* < 0.01).

### Expression of genes related to lipid metabolism and inflammation in WAT

**Figure 6:**
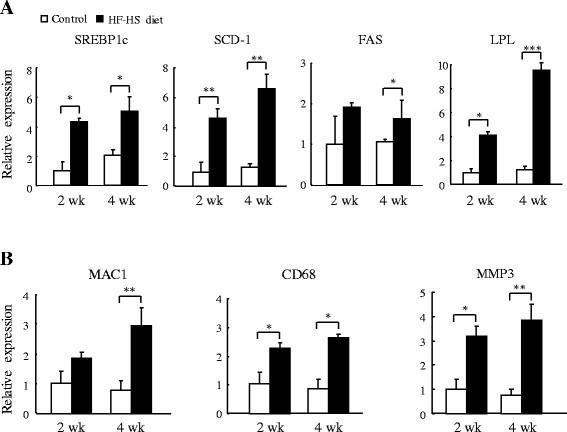
**Effect of the HF–HS diet on WAT genes involved in lipogenesis and inflammation.** (**A**) mRNA expression of genes related to lipogenesis: *SREBP1c*, *SCD-1*, and *FAS*, and of the lipolysis-related gene, *LPL*. (**B**) mRNA expression of genes related to inflammation: *MAC1*, *CD68*, and *MMP3*. HF–HS diet: high fat–high sucrose diet. C57BL/6 J mice were fed standard chow diet or the HF–HS diet (milk fat 21% and sucrose 34%) for 2 or 4 weeks. Values are the mean ± SE and are relative to control values, which were set at 1.0. n = 6. * *P* < 0.05, ** *P* < 0.01, *** *P* < 0.001.

As shown in Figure 6A, the HF–HS diet rapidly upregulated the expression of genes involved in lipogenesis in mesenteric WAT. At the respective weeks 2 and 4, the HF–HS diet increased mRNA expression of *SREBP1c* by 3.3 fold and 1.4 fold (*P* < 0.05 for both), that of *SCD-1* by 3.6 fold and 4.0 fold (*P* < 0.01 for both), and that of *FAS* by 0.9 fold (not significant) and 0.5 fold (*P* < 0.05). mRNA expression of the gene encoding the lipolytic enzyme LPL was increased by 3.2 fold (*P* < 0.05) and 6.7 fold (*P* < 0.001) by the HF–HS diet at weeks 2 and 4, respectively. Furthermore, compared with the control group, genes related to inflammation were upregulated in animals given the HF–HS diet (Figure 6B). At the respective weeks 2 and 4, the HF–HS diet increased the mRNA expression of macrophage-1 antigen (*MAC1*) by 0.8 fold (not significant) and 2.9 fold (*P* < 0.01), that of *CD68* by 1.2 fold and 2.1 fold (*P* < 0.05 for both), and that of matrix metalloprotease 3 (*MMP3*) by 2.2 fold (*P* < 0.05) and 4.1 fold (*P* < 0.01).

## Discussion

In this study, we demonstrated that short-term feeding of the HF–HS diet caused gains in body and WAT weight and hepatic steatosis as early as 2 weeks. The calories in the HF–HS diet were 26% higher than those in the standard chow (control). Although there was no difference in food intake between animals given the control and HF–HS diet, daily caloric intake was increased by the HF–HS diet. Thus, rapid onset of visceral obesity and fatty liver may occur with intake of a high-calorie diet that is high in fat and sucrose.

Diet-induced obesity is largely caused by disorders of fat metabolism, resulting in a massive accumulation of fat in various tissues. Lipid and energy metabolism are regulated by a complex network of signalling processes, and we therefore investigated mRNA expression of key genes regulating lipid metabolism such as *LPLSREBP1cSCD-1FASLXRα*, and *PPARγ*. The HF–HS diet upregulated WAT and liver *LPL* mRNA expression. The lipolytic enzyme LPL mediates uptake of circulating lipid into peripheral organs, and it is the primary enzyme responsible for chylomicron- and very low–density lipoprotein–triglyceride lipolysis [14]. Excessive circulating lipids from the HF–HS diet may have stimulated *LPL* overexpression, and it has been demonstrated that tissue-specific overexpression of *LPL* in skeletal muscle and liver increases cellular stores of triglycerides and leads to insulin resistance [15]. mRNA levels of genes encoding lipogenic proteins such as *SREBP1cSCD-1*, and *FAS* were increased similarly by the HF–HS diet at weeks 2 and 4, suggesting a rapid effect of the HF–HS diet on *de novo* lipogenesis. The lipogenic transcription factor SREBP1c plays a crucial role in regulating fatty acid synthesis, and SREBP1c–responsive major genes include *acetyl CoA carboxylaseFAS*, which encodes a rate-limiting enzyme in *de novo* fatty acid biosynthesis to produce palmitic acid, *SCD-1*, which converts stearic acid to oleic acid, and *glycerol-3-phosphate acyltransferase*, which encodes the first committed enzyme in triglyceride and phospholipid synthesis [16,17]. The HF–HS diet contained 34% sucrose, and there is increasing evidence that fructose and glucose, the components of sucrose, contribute to the regulation of lipid metabolism partially by acting as inducers of *SREBP1*c and *SCD-1*[18,19]. Furthermore, LXR is also a potent stimulator of fatty acid and triglyceride synthesis, and activation of LXRα in liver is closely related to the development of non-alcoholic fatty liver disease [20,21]. LXR binds to DNA as a heterodimer with the retinoid X receptor (RXR) and acts as a key transcriptional regulator of lipid metabolism, mediated largely by LXR-dependent hepatic expression of *SREBP-1c* and its downstream target genes [22,23]. LXR agonists induce the expression of genes associated with fatty acid biosynthesis [24]. In the current study, a rapid increase in *LXRα* mRNA expression in liver was observed after feeding the HF–HS diet. It has been shown that glucose binds to and stimulates the transcriptional activity of LXR, and glucose induces expression of LXR target genes with an efficacy similar to that of the dietary LXR ligands, oxysterols, suggesting that glucose is an endogenous LXR ligand [25]. In addition, hepatic PPARγ has been reported to be closely related to hepatosteatosis, and several studies using diabetic or obese murine models have shown that *PPARγ* expression is elevated in liver [26-29]. Liver-specific disruption of *PPARγ* in diabetic mice dramatically decreases hepatic triglyceride levels and systemically perturbs insulin signalling [30]. Thus, a role for PPARγ as an inducer of steatosis in hepatocytes has been suggested, and our results also show upregulation of *PPARγ* mRNA and its target gene *CD36* in liver by the HF–HS diet. Collectively, the rapid response of lipid metabolism–related genes in response to a diet high in fat and sucrose is likely to contribute to the rapid effect of such a diet on obesity-associated hepatosteatosis.

After feeding with the HF–HS diet, concomitant with downregulation of *IRS2Akt2*, and *AMPK* that are involved in the insulin signalling pathway in liver, plasma insulin levels were elevated similarly at weeks 2 and 4, suggesting that impaired insulin signalling was rapidly triggered in response to the HF–HS diet. Increased adiposity and hepatic lipid accumulation are closely associated with insulin resistance [31]. The intracellular accumulation of diglycerides leads to activation of protein kinase C, which in turn decreases insulin-stimulated IRS-1/IRS-2 tyrosine phosphorylation, phosphoinositide-3 kinase activation, and downstream insulin signalling [32]. Chronic exposure to fructose causes hyperinsulinaemia and obesity through altered mechanisms that include the effect of fructose on ATP depletion and uric acid generation [33], increased circulating C-peptide levels that are often associated with insulin resistance [34], involvement of the fructose transporter GLUT5 that shows significantly higher expression levels in young Zucker obese rats compared to lean controls [35], and the hexosamine hypothesis, in which hexosamine flux is thought to be involved in regulating glucose pathways [36].

The HF–HS diet increased the expression of the macrophage marker *MAC1* as well as *CD68* and the inflammatory marker *MMP3* in WAT, and it significantly elevated plasma resistin levels. Accumulating evidence has shown that obesity-associated insulin resistance is related to adipose tissue dysfunction that is aggravated by the development of chronic mild inflammation [37,38]. Infiltration of macrophages into the hypertrophic adipose tissue promotes inflammation and introduces pro-inflammatory adipokines into circulation and insulin-targeted tissues [39]. Resistin, a pro-inflammatory adipokine found in inflammatory zone 3 (Fizz3) and adipocyte-specific secretory factor, has been shown to assert its effects on the inflammatory pathway and impair insulin sensitivity [40,41]. Circulating levels of resistin are increased in obesity, and an increase in serum resistin level has been shown to induce insulin resistance in rodents, suggesting that hyperresistinaemia has a causal relationship with obesity-associated insulin resistance [42-44]. Therefore, the rapid elevated expression of inflammation-related genes in WAT with the HF–HS diet may be associated with increased plasma resistin levels, which in turn may contribute to impaired insulin signalling and compensatory hyperinsulinaemia.

The HF–HS diet caused hypercholesterolaemia as early as 2 weeks, but no difference was found in plasma triglyceride and free fatty acid levels between the control and HF–HS groups. A similar plasma lipid profile was observed in mice fed a high-fat diet [45]. A wealth of evidence has demonstrated the importance of SREBP2 in regulating hepatic cholesterol homeostasis. SREBP2 upregulates key cholesterogenic genes, such as those encoding *LDLR* and *HMGCR*[46]. The HF–HS diet did not alter the mRNA expression of these genes that play crucial roles in regulating cholesterol synthesis, suggesting a minor role for cholesterogenic genes in the hypercholesterolaemia induced by the HF–HS diet. Furthermore, the absence of changes in plasma lipid levels with the HF–HS diet may possibly be explained by elevated plasma triglyceride clearance that is partially due to increased expression of *LPL* and *CD36* in liver. In addition to LPL, CD36 also influences the uptake of circulating fatty acids. CD36 is an 88-kDa glycoprotein located at the plasma membrane that plays a key role in enhancing fatty acid uptake into cells. *CD36* overexpression in human hepatoma cells increases the uptake of fatty acids, whereas a *CD36* deficiency significantly impairs fatty acid uptake by peripheral organs such as heart, skeletal muscle, and adipose tissue in rodents [47,48].

## Conclusions

In conclusion, the present study demonstrated that ingestion of a diet high in fat and sucrose by mice significantly elevated WAT weights, hepatic steatosis, and plasma insulin levels as early as 2 weeks. Expression of genes involved in the multiple steps of lipid accumulation and inflammation in liver and WAT increased rapidly in response to the HF–HS diet, and expression of these gene products may have contributed to the rapid accumulation of lipids in these organs and may have further impaired hepatic insulin signalling, leading to compensatory hyperinsulinaemia.

## Abbreviations

AMPK, 5' adenosine monophosphate-activated protein kinase; Akt2, Protein kinase B beta; CD36, CD36 antigen; CD68, CD68 antigen; FAS, Fatty acid synthase; HF–HS diet, High fat–high sucrose diet; HMGCR, 3-hydroxy-3-methylglutaryl-coenzyme A reductase; IRS2, Insulin receptor substrate 2; LDLR, Low-density lipoprotein receptor; LPL, Lipoprotein lipase; LXRα, Liver X receptor alpha; MAC1, Macrophage-1 antigen; MMP3, Matrix metalloprotease 3; PPARγ, Peroxisome proliferator activated receptor gamma; QPCR, Quantitative polymerase chain reaction; SCD-1, Stearoyl CoA desaturase-1; SREBP, Sterol regulatory element binding protein; WAT, White adipose tissue.

## Competing interests

The authors declare that they have no competing interests.

## Authors’ contributions

Conceived and designed the experiments: ZHY, HM, JT, and MK. Performed the experiments and analyzed the data: ZHY and HM. Wrote the paper: ZHY. All authors read and approved the final manuscript.
